# A Hybrid Machine Learning Model for Dynamic Level Detection of Lead-Acid Battery Electrolyte Using a Flat-Plate Capacitive Sensor

**DOI:** 10.3390/s26020361

**Published:** 2026-01-06

**Authors:** Shuai Huang, Weikang Zhang, Weiwei Zhang, Zhihui Ni, Lifeng Bian, Jiawen Liu, Peng Yue, Peng Xu

**Affiliations:** 1 School of Energy and Environment, Southeast University, Nanjing 210096, China; 220234983@seu.edu.cn (S.H.); 220224601@seu.edu.cn (J.L.); 2 School of Energy Science and Engineering, Nanjing Tech University, Nanjing 211816, China; zhangweikang@njtech.edu.cn (W.Z.); yuepeng2025@njtech.edu.cn (P.Y.); 3 The 6th Academy of Aerospace Science and Industry Corp in China, Hohhot 010010, China; zww_ncepu@163.com; 4 Inner Mongolia Aerospace Power Machinery Testing Institute, Hohhot 010010, China; nizhihui601@163.com (Z.N.); bianlf2009@163.com (L.B.)

**Keywords:** capacitive method liquid level measurement, residual liquid film adhesion layer effect, nondestructive evaluation, polynomial feature generation, long short-term memory, hybrid deep learning model

## Abstract

Abnormal electrolyte levels can lead to failures in lead-acid batteries. The capacitive method, as a non-invasive liquid level inspection technique, can be applied to the nondestructive detection of electrolyte level abnormalities in lead-acid batteries. However, due to the high viscosity of sulfuric acid in lead-acid batteries, residual liquid films are easily adhered to the tube walls during rapid liquid level drops, resulting in significant dynamic measurement errors in capacitive methods. To eliminate dynamic measurement errors caused by residual liquid film adhesion, this study proposes a hybrid deep learning model—Poly-LSTM. This model combines polynomial feature generation with a Long Short-Term Memory (LSTM) network. First, polynomial features are generated to explicitly capture the complex nonlinear and coupling effects in the sensor inputs. Subsequently, the LSTM network processes these features to model their temporal dependencies. Finally, the time information encoded by the LSTM is used to generate accurate liquid level predictions. Experimental results show that this method outperforms other comparative models in terms of liquid level estimation accuracy. At a rapid drop rate of 0.12 mm/s, the average absolute error (MAE) is 0.5319 mm, the root mean square error (RMSE) is 0.7180 mm, and the mean absolute percentage error (MAPE) is 0.1320%.

## 1. Introduction

At present, lead-acid batteries have become a widely used rechargeable battery technology in the world because of their good safety, low cost, and excellent recyclability [1]. The electrolyte liquid level of the lead-acid battery is a critical state parameter of the lead-acid battery. During the long-term storage of lead-acid batteries, the batteries may fail due to reasons such as electrolyte leakage. If the liquid level of the battery electrolyte is too low, it will cause faults and even irreversible damage [2]. In industrial applications, large groups of lead-acid batteries are commonly used. A failure in one cell of a battery group, caused by electrolyte leakage, can directly affect the charging current of other batteries in series within the same group, thereby accelerating the failure of the entire battery set. Currently, nuclear power plants and data centers mainly rely on regular inspections by staff to visually check whether the liquid levels are within the acceptable range. This method is highly inefficient. Therefore, online real-time monitoring of the electrolyte level in each individual lead-acid battery can effectively enhance the reliability of backup power systems in industrial scenarios, which is crucial for failure prevention in safety-critical applications. Current liquid level monitoring methods are mainly divided into contact type and non-contact type [3]. For contact methods, common techniques include float level gauges [4], conductive level sensors [5], and pressure sensors [6]. For non-contact methods, the main technologies include ultrasonic level sensors [7], laser level sensors [8], fiber optic sensors [9], capacitive level sensors [10], and frequency-modulated millimeter wave sensors [11]. In industrial settings such as nuclear power plants and large data centers, contact measurement of lead-acid battery electrolyte is not permitted; online inspection systems must be capable of non-contact measurement. Among various non-contact measurement methods, ultrasonic level sensors and laser level sensors are typically installed inside the lead-acid battery container, which is destructive to battery manufacturers who usually refuse to install them. Therefore, currently only capacitive level sensors and frequency-modulated millimeter wave sensors can be retrofitted to modify existing battery pack detection systems. However, in industrial applications, lead-acid batteries are typically arranged in close proximity, with only a few millimeters of gap between individual cells. As a result, frequency-modulated millimeter wave sensors, due to their large size, cannot be effectively installed in practical application scenarios. In contrast, capacitive level sensors, with electrode thicknesses as thin as 0.1 mm, can effectively avoid the aforementioned installation issues and hold promising prospects for practical applications.

Capacitive liquid level measurement is a mature and widely used technology. Jayalaxmi and Santhosh [12] proposed and implemented a new type of helical electrode capacitive liquid level sensor. The results showed that the helical electrode had improvements in indicators such as sensitivity and full-scale response time. Bera et al. [13] addressed the nonlinearity and temperature dependency issues inherent in capacitance-type level sensors, especially when measuring liquids with low dielectric constants.

However, the above-mentioned capacitive liquid level measurement method usually has a large measurement error in the dynamic measurement of the liquid level of lead-acid battery electrolyte. This is mainly because the sulfuric acid electrolyte in lead-acid batteries has a high viscosity, and as the electrolyte descends, it forms a liquid film on the container wall due to adhesion. These liquid films have a higher capacitance value compared to pure air, which leads to the measured capacitance value being larger than the true value, thus affecting the accuracy of traditional capacitance-level linear models. If this situation occurs in high-safety-requirement environments such as nuclear power plants, false level alarms could trigger unnecessary safety actions, causing interruptions to industrial production processes.

In response to the above problems, some researchers have indeed noticed the phenomenon of residual liquid film adhesion layer effect in dynamic liquid level measurement and have carried out research on it. Jayalaxmi and Santhosh [14] analyzed and compared the dynamic response characteristics and steady-state response characteristics of planar, helical, and cylindrical capacitive sensors when the liquid flowed in and out at three different rates. They found that the helical type had the fastest response time. They also found that the response times of each structure under the three different outflow rates were not the same, which was caused by the hysteresis effect resulting from the residual liquid film adhesion. But they did not analyze the reasons for the differences in response conditions among various structures under different inflow and outflow rates. Nisio [15] designed three capacitive sensor plates with different structures and used delay times of 0 s, 5 s, and 10 s, respectively, after the liquid level stabilized to deal with the residual liquid film adhesion layer effect. But using the delay waiting method to deal with the residual liquid film adhesion layer effect not only affects the real-time performance of online measurement, but also affects the accuracy of measurement results due to the uneven migration of the liquid film.

To address this issue, this study establishes a hybrid machine learning model to resolve the dynamic measurement error caused by the residual liquid film adhesion layer effect. Experiments show that the interference of the residual liquid film on the measurement is affected by the liquid level drop rate. The liquid level drop rate is related to the capacitance change within each sampling interval. Therefore, the problem can be transformed into establishing a model with multidimensional features as input and liquid level prediction as output.

In recent years, with the rapid development of machine learning and deep learning, machine learning models such as recurrent neural networks (RNN) and long short-term memory networks (LSTM) have been widely used in data modeling and fault detection. However, in practical applications, neural networks such as RNN and LSTM usually require a large amount of training data to fully learn effective patterns. When the amount of data is small or the features are insufficient, the neural network is prone to overfitting, resulting in poor model generalization ability [16]. Therefore, in complex nonlinear coupling scenarios, it is usually difficult to capture some implicit nonlinear relationships between complex coupling features by using only a single neural network model [17].

To address these issues, this study builds the Poly-LSTM model which combines LSTM (long short-term memory) with Poly (polynomial feature generation) to compensate for the deficiencies of a single neural network. Polynomial feature generation is a powerful feature engineering technique, and its core advantage lies in its ability to explicitly capture and model the hidden nonlinear relationships within the data as well as the interaction effects among different features [18]. By using polynomial feature generation, these high-order terms and interaction terms can be automatically created, transforming these complex nonlinear patterns from an imperceptible implicit state to a clear explicit state. This enables LSTM to directly receive these pre-computed nonlinear features with greater information content, thereby significantly reducing its learning burden and allowing it to focus more efficiently on capturing the dynamic patterns of these nonlinear features over time. Ultimately, this combination not only significantly improves the model’s prediction accuracy but also provides stronger transparency and physical interpretability for the model’s decisions.

The remainder of this paper is organized as follows. Section 2 describes the experimental setup and analyzes the effect of the residual liquid film on dynamic measurements, which demonstrates the limitations of the traditional static linear model. Section 3 presents the proposed Poly-LSTM methodology in detail, including the multidimensional feature extraction and the architecture of the integrated learning model. Section 4 presents the experimental results, providing a comparative analysis of the proposed model against baseline models under various and variable liquid level descent rates. Finally, Section 5 concludes the paper and discusses future work.

## 2. System Design and Characterization of the Residual Film Effect

**Figure 1:**
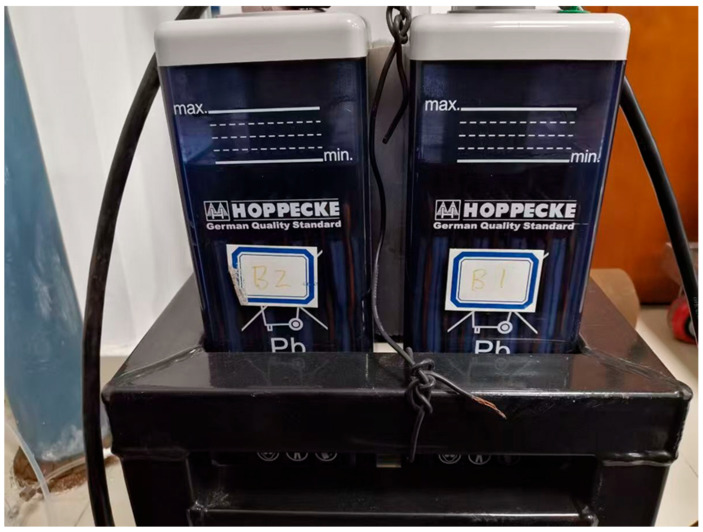
Upper and lower limit markings for the liquid level of lead-acid batteries.

The electrolyte level of a lead-acid battery has upper and lower limits. The upper and lower limits of the liquid level are marked on the shell of the lead-acid battery, as shown in Figure 1.

**Figure 2:**
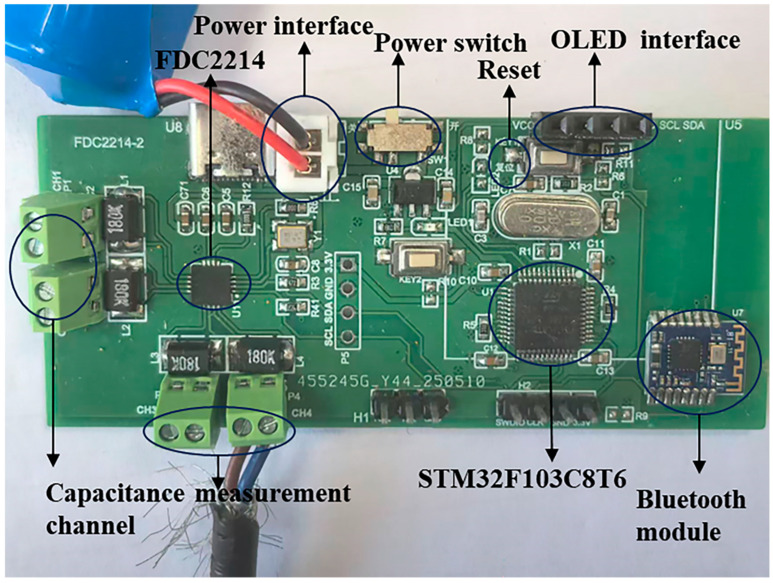
Physical diagram of the capacitive sensor.

**Figure 3:**
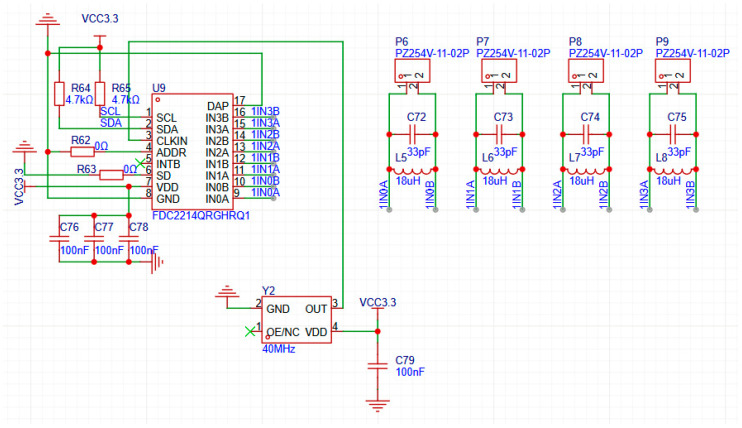
FDC2214 multi-channel capacitive sensing circuit.

In order to achieve high-precision capture and stable measurement of minute capacitance changes, this research has designed a hardware system that integrates a microcontroller, a dedicated capacitance detection chip, and a data communication module. The aim is to provide reliable capacitance measurement data for subsequent dynamic liquid level monitoring and the verification of intelligent algorithms. The main control board of this system selects the STM32F103C8T6 microcontroller from STMicroelectronics as the main control chip of the minimal system board and uses the FDC2214 capacitance measurement chip to measure the precise capacitance value. The physical diagram of the capacitive sensor and the circuit diagram of the FDC2214 capacitive measurement chip are, respectively, shown in Figure 2 and Figure 3.

Figure 3 shows the implementation of the sensor front-end circuit. The core of this circuit is U9 (FDC2214), a multi-channel capacitance-to-digital converter. Its working principle is to measure the resonant frequencies of four independent LC resonant circuits. These circuits are composed of inductors L5–L8, capacitors C72–C75, and external sensing electrodes connected to P6–P9. When the target to be measured approaches the sensing electrodes, it causes a slight change in the sensor capacitance, which in turn leads to a deviation in the resonant frequency of the LC circuits. The FDC2214 chip precisely detects this frequency deviation and converts it into a digital signal. Additionally, Y2 is a 40 MHz active crystal oscillator, providing a high-precision clock reference for the FDC2214; the SCL and SDA pins and the pull-up resistors R64 and R65 form the I^2^C communication interface for data transmission with the microcontroller; C76 to C79 are power bypass capacitors used to filter out noise and ensure the stable operation of the chip.

For each channel, the FDC2214 measures the oscillation frequency of an LC resonant circuit composed of an external inductor and the capacitance of the sensor to be tested, and converts this frequency into a high-resolution digital value. The relevant calculation formulas are as shown in the official technical manual of Texas Instruments [19]:

(1) $$f_{sensor}=\frac{1}{2\pi \sqrt{L\times C_{sensor}}}$$

(2) $$data=\frac{f_{sensor}\times 2^{28}}{f_{ref}}$$

$f_{sensor}$ is the oscillation frequency of this LC resonant circuit. $f_{ref}$ is the reference clock frequency. $C_{sensor}$ is the capacitance to be measured. L is 18 μH in this chip. The core function of the FDC2214 chip is to precisely measure the oscillation frequency of this LC resonant circuit. The chip will measure the ratio of the sensor frequency $f_{sensor}$ to the reference frequency $f_{ref}$, and then convert this ratio into a digital value for output data. After the microcontroller reads this digital value data from FDC2214, it can calculate the current oscillation frequency $f_{sensor}$ of the sensor based on the known $f_{ref}$. By combining the LC resonant frequency formula, the known inductance value L and the other fixed parallel capacitance values in the circuit, the current value of the sensor capacitance $C_{sensor}$ can be calculated. 

**Figure 4:**
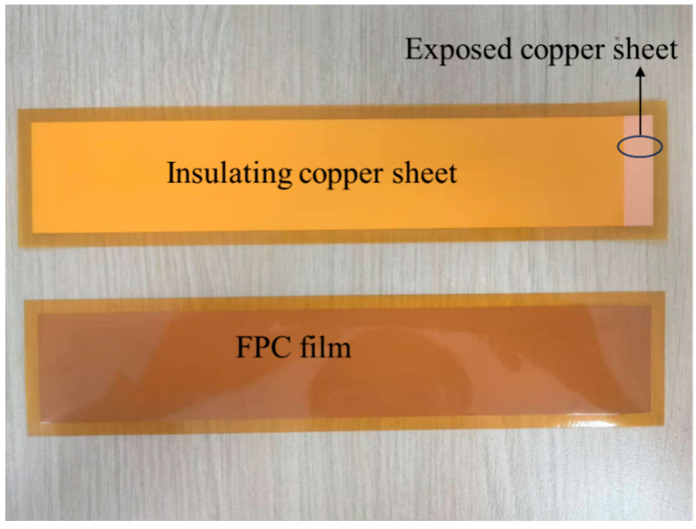
Physical pictures of the positive and negative sides of the electrode.

In this study, copper sheets were used as the electrode material. To provide insulation protection, the flexible printed circuit (FPC) technology was adopted. The capacitor plates were fabricated by covering the copper sheets with the FPC material itself. Specifically, the conductive copper foil layer of the sensor was integrated into the multi-layer structure of the FPC, and its exposed surface was protected and encapsulated by the insulating film of the FPC. The physical diagrams of the front and back sides are shown in Figure 4. Most of the copper sheet areas were covered by the FPC insulating material, and a small part of the exposed bare copper sheet was exposed at the ends to facilitate welding with the wires. Such a design can provide excellent electrical insulation and physical protection. At the same time, the flexibility of the FPC enables it to better fit different shapes of container shells, ensuring the stability of the measurement. The size of the electrode is 235 mm in length, 50 mm in width, and 0.1 mm in thickness. The exposed part of the copper sheet is 10 mm long.

**Figure 5:**
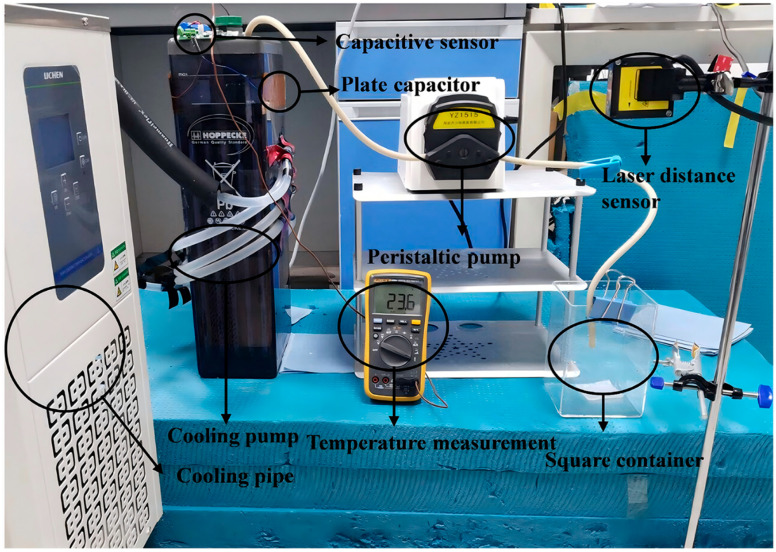
Experimental equipment layout.

**Figure 6:**
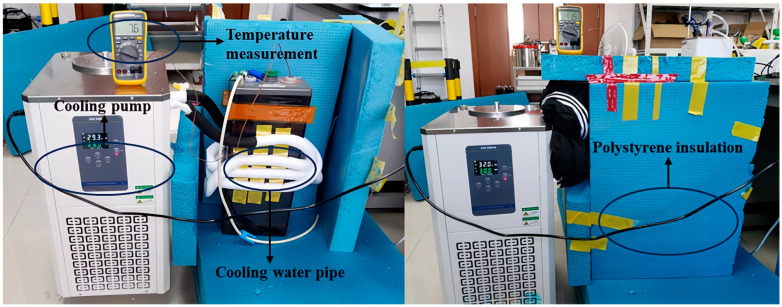
Cooling and insulation of lead-acid batteries.

**Figure 7:**
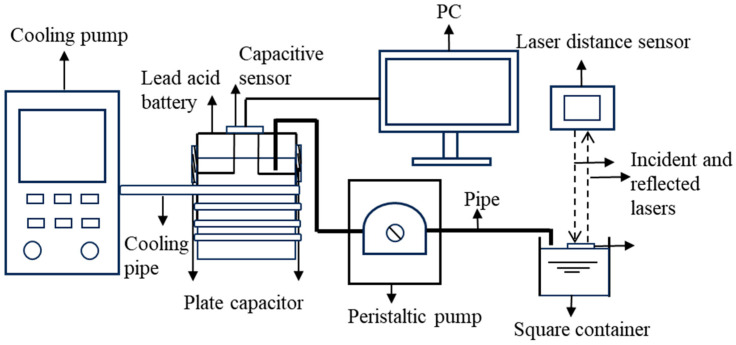
Experimental equipment schematic diagram.

The experimental equipment layout is shown in Figure 5. Figure 6 shows the cooling and insulation of lead-acid batteries for the verification of the temperature robustness of the model, and Figure 7 is the schematic diagram of the experimental equipment. The two capacitive plates attached to the vertical surface of the container served as the positive and negative electrodes of the capacitor. The capacitor plate covered the upper and lower limits of the electrolyte.

The actual industrial lead-acid batteries used for observation or operation typically have a very small top opening area. This structural feature makes it impossible to adopt the method where a floating sheet is inserted and a laser displacement sensor is used for high-precision liquid level reference measurement. To overcome these problems, this experiment uses the indirect method. The extracted electrolyte was sent into the square container. A laser distance sensor was mounted above the container and directs the laser beam onto a floating Teflon sheet on the liquid surface, measuring to an accuracy of 0.1 μm. Based on the measured level of the sulfuric acid, its volume can be accurately calculated. Then, by dividing this volume by the base area of the lead-acid battery, the electrolyte level of the lead-acid battery can be precisely determined. This indirect measurement method can achieve precise liquid level measurement without disassembling the battery. The peristaltic pump is used to precisely control the speed at which the electrolyte is extracted.

In order to rigorously evaluate the robustness of the proposed deep learning model under different thermal conditions, a dedicated temperature control system using the low-temperature cooling liquid circulation pump was constructed, as shown in Figure 6. The cooling mechanism relies on contact heat conduction. Flexible cooling tubes are tightly wound around the casing of the lead-acid battery. 95% mass fraction ethanol was selected as the coolant to ensure stable flow and heat transfer efficiency at low temperatures. Thermocouples are used to monitor the temperature of the battery terminals in real time.

During the experiment, the cooling and circulation functions of the pump were activated, and heat was continuously extracted from the battery. To minimize heat exchange with the surrounding environment and ensure uniform cooling, the entire battery component was wrapped in polystyrene insulation material. A thermocouple firmly connected to the top terminal of the battery was used to monitor the battery temperature in real time.

During each experimental run, two primary data streams were collected simultaneously. The capacitance value was measured in pico-Farads by the capacitive sensor, and the true liquid level was measured in millimeters by the interpolation calculation method for starting and ending liquid levels. The sampling time is 200 ms. A custom data acquisition program developed in PyCharm 2020 Community Edition was used to log the data from both instruments to a host computer, and the environmental temperature was maintained at 20 °C.

Regarding the capillary action, while a meniscus is naturally formed at the liquid–wall interface, its effect is considered a systematic component of the measurement in the setup. The true liquid level was measured at the center of the container, away from the immediate wall effects. The capacitance sensor, which integrates the electric field over the entire wetted plate area, inherently includes the effect of the meniscus. As the data-driven model is trained to learn the direct mapping from the total measured capacitance which includes all systematic effects like capillarity to the true bulk level, the consistent influence of the capillary action is implicitly learned and compensated for by the model during the training process.

To build a comprehensive dataset for model training and evaluation, experiments were conducted under four distinct scenarios by changing the rotational speed of the peristaltic pump to 15, 30, 60, and 100 rpm. The experimental run performed at a very slow descent rate; 15 rpm was used to establish a quasi-static baseline. It is worth noting that the extremely fast decreasing rate corresponding to sudden structural failures such as severe shell rupture was not included in the detailed estimation analysis. Such emergency situations fall within the scope of fault safety protection logic rather than continuous liquid level tracking. This research aims to address the challenge of monitoring slow and imperceptible liquid level drops to prevent minor abnormalities from escalating into major safety accidents. The experimental runs performed at three constant descent rates were used to train and test the machine learning (ML) model. Meanwhile, this experiment also conducted variable rates experiment by changing the rotational speed of the peristaltic pump to simulate real industrial scenarios to verify the robustness of the model.

To comprehensively evaluate the robustness of the proposed model against environmental temperature variations, experiments were conducted under four distinct thermal conditions: 5 °C, 15 °C, 20 °C, and 25 °C. The standard temperature for lead-acid batteries is 20 °C. The ideal operating temperature range is 20 °C ± 5 °C [20]. The 5 °C is set to test the robustness of the model under extreme conditions. The test temperature should not be too high or too low, because, based on the standard of 20 °C ± 5 °C, higher temperatures will shorten the lifespan of the equipment, while lower temperatures will reduce the available capacity [20]. The dataset collected at the standard temperature for lead-acid batteries, 20 °C, was utilized for model training, hyperparameter tuning, and initial performance validation. In contrast, the datasets obtained at other temperatures (5 °C, 15 °C, and 25 °C) were kept entirely separate from the training process. These datasets served exclusively as independent test sets to assess the model’s generalization ability and to verify its stability when operating in different thermal environments.

This process resulted in a total dataset containing approximately 42,000 sample points. The detailed composition of the dataset is provided in Table 1.

**Figure 8:**
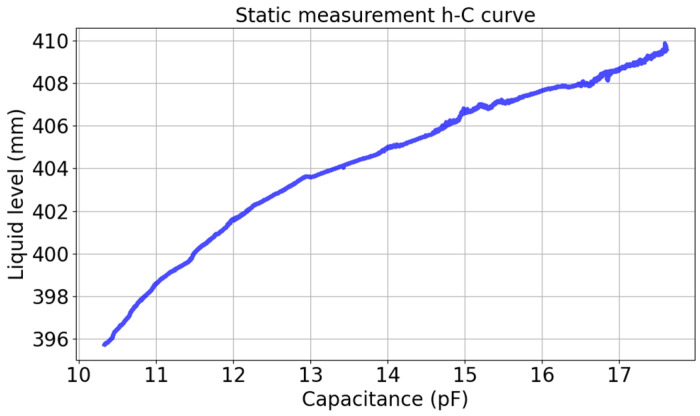
Static measurement relationship diagram.

The relationship between capacitance and liquid level is as follows [10]:

(3) $$C_{1}=\frac{\in_{0}\in_{r}A_{1}}{d}=\frac{\in_{0}\in_{r1}a\left(l_{1}-h\right)}{d}$$

(4) $$C_{2}=\frac{\in_{0}\in_{r}A_{2}}{d}=\frac{\in_{0}\in_{r2}a\left({h-l}_{2}\right)}{d}$$

(5) $$\begin{array}{cc} C & =C_{1}+C_{2}\\ & =\frac{{(\in }_{0}\in_{r2}a-\in_{0}\in_{r1}a)h}{d}\\ & \;+\frac{\in_{0}\in_{r1}al_{1}-\in_{0}\in_{r2}al_{2}}{d} \end{array}$$

where $C,\;C_{1},\;C_{2}$ are the total capacitance, capacitance of the air, and capacitance of the liquid. $\in_{0},\in_{r1\;},\in_{r2}$ are the vacuum permittivity, relative permittivity of the air, and relative permittivity of the liquid. $A_{1}\;and\;A_{2}$ are the area of the capacitor plates in contact with air and area of the capacitor plates in contact with liquid. $l_{1}\;and\;l_{2}$ are the height of the top of the plates and height of the bottom of the plates. h is the liquid level height. d is the distance between the plates. a is the width of the plates. From Equation (5) it can be seen that the liquid level has a good linear relationship with capacitance. When other conditions remain constant, all parameters except C and h are constants. So, Equation (5) can be simplified to a linear model:

(6) h=kC+b

where k and b are constant. This linear relation is suitable for static measurement. This experiment controlled the peristaltic pump to run at a very slow rates of 0.06 mm/s to simulate static measurement. Since this condition represents a stable, near-linear physical relationship, this single, continuous data acquisition pass was sufficient to accurately fit the baseline static linear model. The relationship between liquid level and capacitance is shown in Figure 8. The figure shows an approximately linear relationship between the level and capacitance. Using the least square method to fit the linear relation, the relation is as follows:

(7) h=2.0489C+375.8935

**Figure 9:**
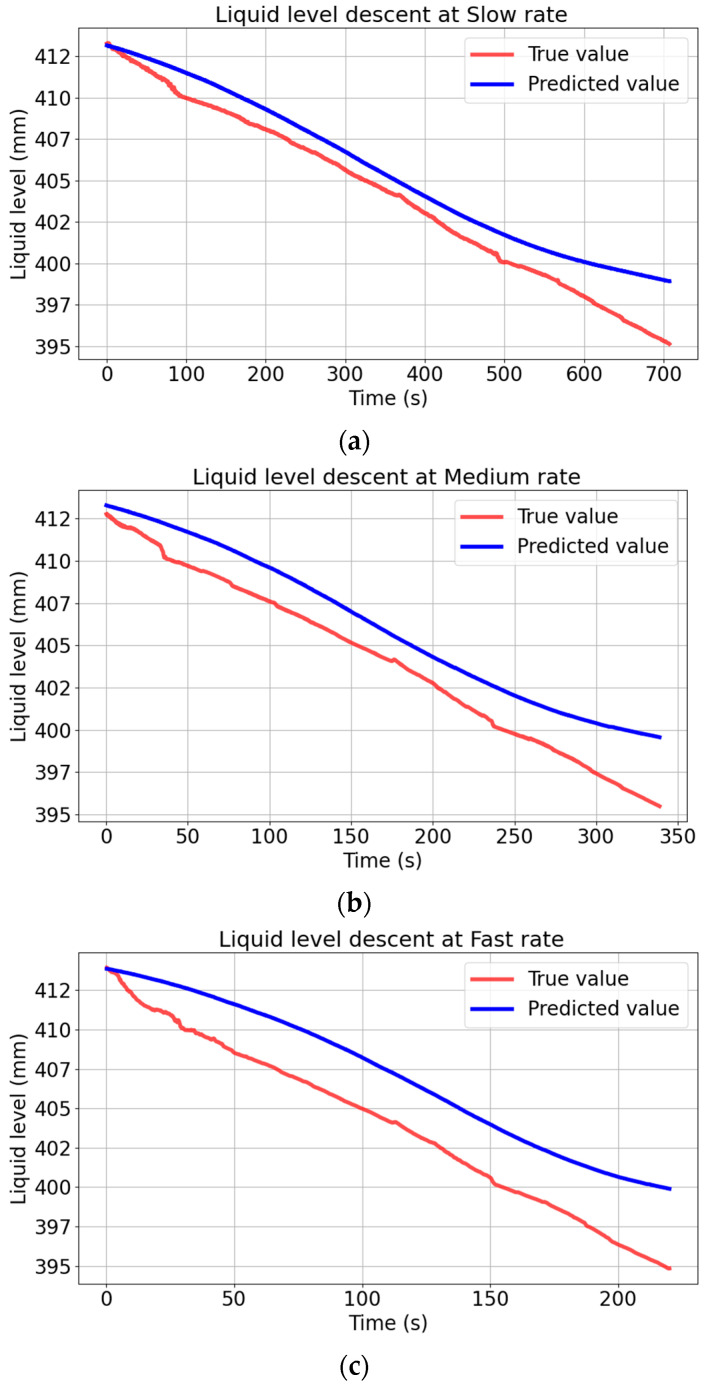
Comparison between the static linear model prediction and the true value at different descent rates. (**a**) Comparison between the static linear model prediction and the true value at slow rate. (**b**) Comparison between the static linear model prediction and the true value at medium rate. (**c**) Comparison between the static linear model prediction and the true value at fast rate.

This model is applied to liquid level measurement with different descent rates, and the results are shown in Figure 9. All the sample points during the descent process were recorded at different liquid level descent rates. Owing to the varying descent rates, the time needed to extract a liquid of the same height differs. Consequently, the scale of the time axis varies.

Figure 9 shows that the static linear model has a good prediction effect for slow descent. However, the deviation between the predicted value curve and the true value curve is larger in the case of fast descent, and the faster it descends, the greater the deviation. In order to test the effect of the static linear model from the evaluation index of the model, this study employed the evaluation indicators from study [21], including the mean absolute error (MAE), root mean square error (RMSE), mean absolute percentage error (MAPE), and coefficient of determination (R^2^). The equations of the evaluation index are given as follows:

(8) $$RMSE=\sqrt{\frac{1}{n}\sum_{i=1}^{n}{\left(y_{i}-\hat{y_{i}}\right)}^{2}}$$

(9) $$MAE=\frac{1}{n}\sum_{i=1}^{n}\left|y_{i}-\hat{y_{i}}\right|$$

(10) $$MAPE=\frac{1}{n}\sum_{i=1}^{n}\left|\frac{y_{i}-\hat{y_{i}}}{y_{i}}\right|\times 100\%$$

(11) $$R^{2}=1-\frac{\sum_{i=1}^{n}{\left(y_{i}-\hat{y_{i}}\right)}^{2}}{\sum_{i=1}^{n}{\left(y_{i}-\bar{y_{i}}\right)}^{2}}$$

The values of indexes are shown in Table 2.

**Figure 10:**
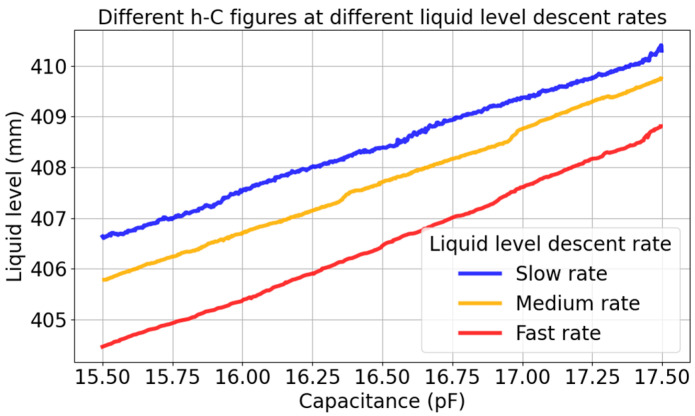
Comparison of level capacitance curves at different descent rates.

Table 2 shows that the MAE, RMSE, and MAPE all increase as the rotate speed increases. This is because, when rapidly descending, the liquid film hanging on the wall does not have enough time to fall, resulting in an increase in the measured capacitance value. As it shows in Figure 10, the same capacitance corresponds to different liquid level at different rates. This error is not allowed in places with high safety requirements such as nuclear power plants. Therefore, the static linear model is not suitable for dynamic fast liquid level descent rates measurement.

## 3. Methodology

**Figure 11:**
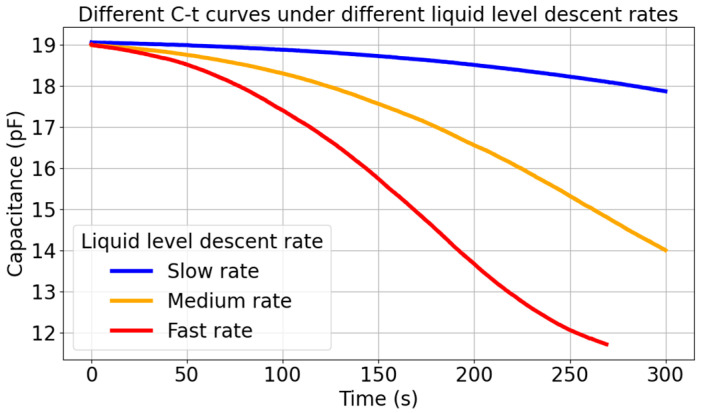
Comparison of capacitance time curves at different descent rates.

The rate of liquid level drop is an important factor affecting capacitance-based liquid level measurement. In order to study the relationship between the rate of liquid level drop and capacitance, we conducted experiments with three different rates of liquid level drop and thus obtained the relationship between the rate of liquid level drop and the rate of capacitance change. Figure 11 shows the relationship between the rate of liquid level drop and the rate of capacitance change.

Figure 11 shows that the liquid level descent rate is related to the capacitance change rate. The faster the liquid level drops, the faster the capacitance changes. Therefore, the features of the model are capacitance and the capacitance change rate, and the label is liquid level.

In this study, the Poly-LSTM model is adopted to compensate for the shortcomings of static models by capturing both explicit nonlinear feature interactions and complex temporal dynamics. First, the polynomial features transformer is used to generate 10-dimensional nonlinear features from the raw inputs, providing a richer, transparent feature representation. This feature sequence is then fed into an LSTM network to encode the temporal dynamics of the liquid film. Finally, the hidden state of the last time step, which serves as a comprehensive summary of the input sequence, is passed through a fully connected output layer to achieve accurate liquid level prediction.

### 3.1. Polynomial Feature Transformation

Traditional linear models have limited capabilities in handling complex nonlinear problems. To explicitly capture the nonlinear interactions between the original features, this study adopted the Polynomial Feature Transformation technique. In the research, the original input data contained two core features: C (capacitance value) and ∆C (capacitance change rate). For each input sample, a feature vector α was generated:

(12) X=[C,∆C]

Since the polynomial terms such as $C^{3}$ are highly sensitive to the scale of the input, before the transformation, the original feature vector X was standardized by Z-score. Subsequently, using a 3rd-degree polynomial with a bias term, the 2-dimensional original feature vector X was expanded into a 10-dimensional new feature vector $X_{ploy}$:

(13) $$X_{ploy}=[1,C,∆C,C^{2},C\cdot ∆C,{∆C}^{2},C^{3},C^{2}\cdot ∆C,C\cdot {∆C}^{2},{∆C}^{3}]$$

This transformation is “white-box”, and each new feature generated has a clear physical interpretation. For example, the term C·∆C represents the interaction between the capacitance value and its change rate, which may contain key information about the dynamic changes in the liquid film. To make these newly generated polynomial features numerically comparable and suitable for the subsequent training of neural networks, the study standardized the 10-dimensional $X_{ploy}$ vector again, obtaining the final feature vector $X_{S}$ [22]:

(14) $$x_{S,j}=\frac{x_{j}^{poly}-\mu_{j}}{\sigma_{j}}$$

(15) $$X_{S}=[x_{S,1},\ldots ,x_{S,10}]$$

Here, $\mu_{j}$ and $\sigma_{j}$ represent the average value and the standard deviation of each dimension feature j (j=1,2…10). In this way, the original 2-dimensional features were explicitly transformed and optimized into 10-dimensional highly nonlinear features, laying the foundation for subsequent time series modeling.

Compared with Kernel PCA (KPCA) which generates uninterpretable black-box features, and Empirical Mode Decomposition (EMD) which suffers from high computational latency due to iterative calculations, the proposed Polynomial Feature Generation offers a lightweight, white-box solution that explicitly models the physical coupling effects with minimal resource consumption.

### 3.2. Sequential Modeling with LSTM

#### 3.2.1. Data Sequencing

To utilize LSTM to capture the dynamic temporal dependence in liquid level measurements, the study must first restructure the data into a sequence. The study set a time window length S. The N consecutive data points $X_{S}=[x_{1},x_{2},\ldots ,x_{N}]$ obtained after the processing in Section 3.1 are divided into N−S+1 overlapping sequence samples. The $i_{th}$ training sample $X_{i}^{seq}$ and its corresponding label $y_{i}$ are defined as

(16) $$X_{i}^{seq}=[x_{i},x_{i+1},...,x_{i+S-1}]$$

(17) $$y_{i}=L_{i+S}$$

where $L_{i+s}$ is the actual liquid level at the i+S time step. Therefore, the task of the model is to predict the liquid level at the current time step based on the 10-dimensional feature sequence of the past S time steps.

#### 3.2.2. LSTM Model

The input sequence is processed by a multi-layer LSTM network. As the network iterates through the sequence step by step, it recursively updates its internal parameters to capture temporal dependencies. Upon processing the last data point in the time window, the LSTM yields a final hidden state, denoted as $h_{S}$. This specific vector $h_{S}$ serves as a comprehensive summary of the entire input sequence, effectively encoding the cumulative dynamic patterns and historical information into a fixed-length high-level feature representation.

Finally, this high-level feature vector $h_{S}$ is passed through a fully connected layer (FC Layer) to generate the final liquid level prediction value $\hat{y}$ via a linear affine transformation [23]:

(18) $$\hat{y}=W_{fc}\cdot h_{S}+b_{fc}$$

where $W_{fc}$ and $b_{fc}$ are the weights and bias of the fully connected layer.

#### 3.2.3. Model Training and Evaluation

The training process of the model adopts the backpropagation algorithm [22]. The mean squared error (MSE) is used as the loss function to evaluate the gap between the predicted value $\hat{y}$ and the true value y. The Adam optimizer is used to update all the learnable parameters in the model including the LSTM layer and the W and b in the final fully connected layer based on the gradients calculated by the loss function. To prevent overfitting, the Dropout mechanism is introduced in the LSTM layer. More importantly, this study uses a diverse dataset containing approximately 28,000 samples collected under various constant and variable descent rates. This data diversity combined with the ability of the time series model forces the model to learn the dynamic physical compensation mechanism behind the liquid film adhesion, rather than simply memorizing the patterns of specific operating conditions, thereby ensuring the robustness and generalization ability of the prediction results.

**Figure 12:**
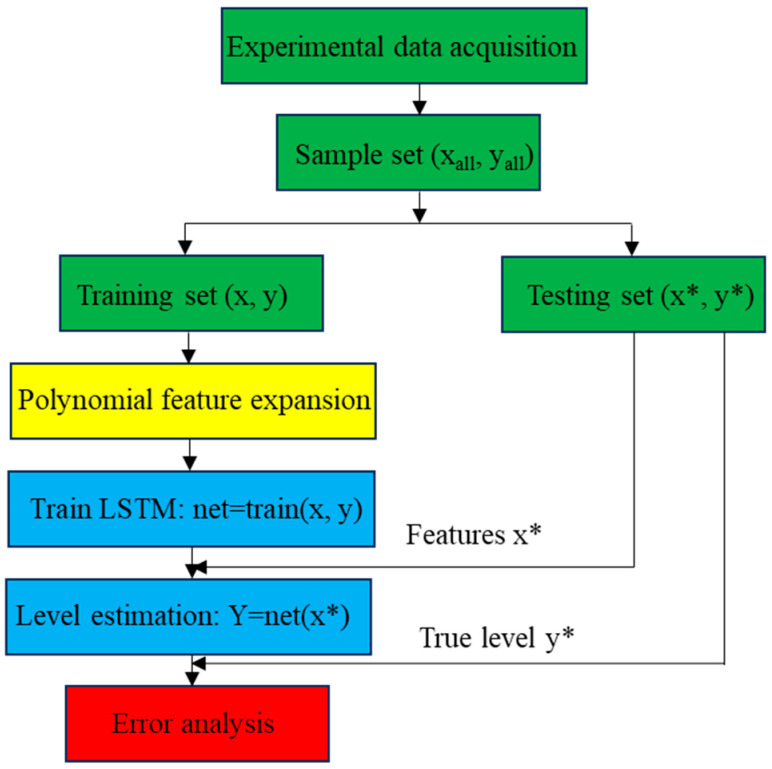
Flow chart of level estimation algorithm.

To sum up, the flow chart of the liquid level estimation algorithm of Poly-LSTM is shown in Figure 12. The “*” indicates that the feature or label belongs to the validation set data.

## 4. Result and Discussion

### 4.1. Determination of Model Hyperparameters

In this study, the dataset is composed of data obtained by repeatedly measuring the different rates of liquid level descent. About 28,000 sample points of different descent rates at an environmental temperature of 20 °C were taken as the training set and test set, ensuring that the data amount is enough for the neural network training. All data were standardized using the Z-Score method before being input into the LSTM model for training as mentioned in Section 3.1. To ensure the optimal performance and methodological rigor of the proposed model, a systematic hyperparameter tuning process was implemented using a grid search approach combined with a 5-fold cross-validation scheme. In the dataset, 75% of the data is designated for training the model, and 25% is designated for model test. All hyperparameter optimization was performed exclusively on the 75% training set to prevent data leakage and ensure an unbiased evaluation on the test set. The grid search explored a predefined space of key hyperparameters. In order to compare the performance of different algorithms, Poly-LSTM, GBDT, MLP, and LSTM were used for comparison. The hyperparameters that need to be adjusted for each model are shown in Table 3. The search spaces select the commonly used values of different hyperparameters in machine learning.

The combination of hyperparameters that yielded the lowest average mean absolute error (MAE) during cross-validation was selected as the optimal configuration. The final model, configured with these optimal settings as presented in Table 4, Table 5, Table 6 and Table 7 was then retrained on the entire training set and its definitive performance was evaluated on the test set.

### 4.2. Validation of the Model for Different Liquid Level Descent Rates

**Figure 13:**
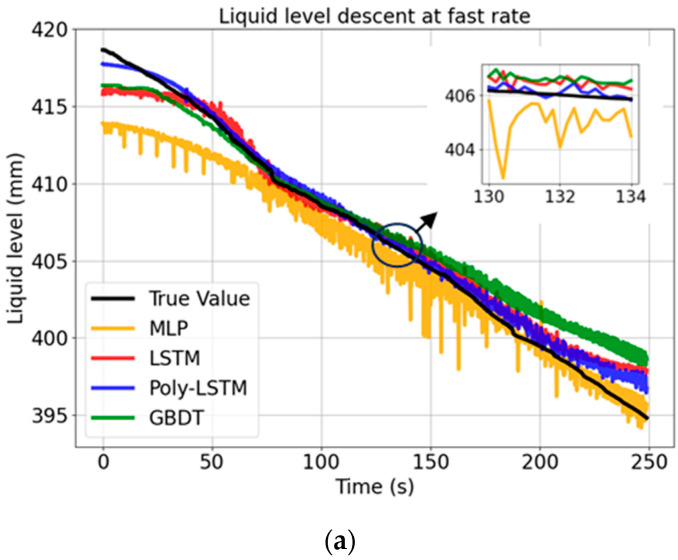

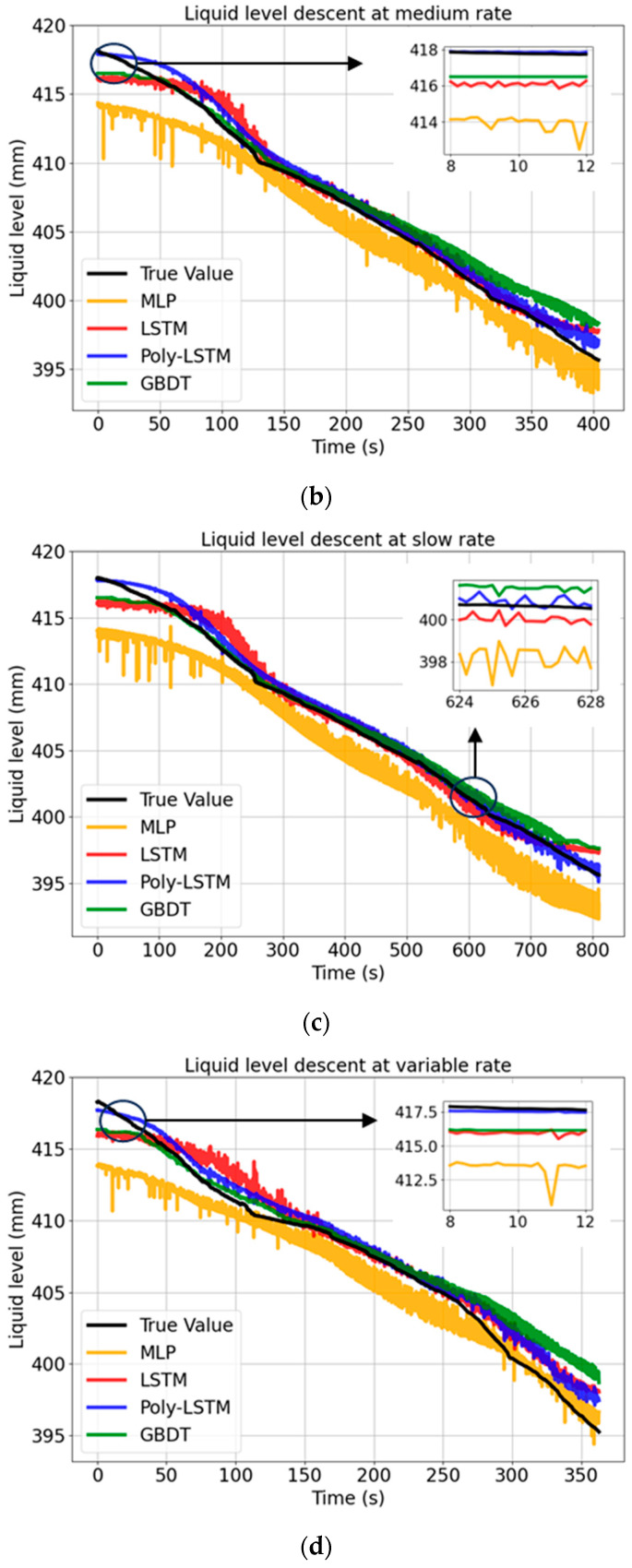
Level estimation results of different models at different rates. (**a**) Level estimation results of different models at slow descent rate. (**b**) Level estimation results of different models at medium descent rate. (**c**) Level estimation results of different models at fast descent rate. (**d**) Level estimation results of different models at variable rate.

The liquid level estimation results are shown in Figure 13. Owing to the varying descent rates, the time needed to extract a liquid of the same height differs. Consequently, the scale of the time axis varies.

Figure 13 shows that the prediction curve of Poly-LSTM is the closest to the real value curve. In contrast, the fluctuation of the MLP model is the most intense. This is mainly because the MLP is highly sensitive to the noise in the original capacitor data; without a dedicated temporal structure or feature engineering, it is prone to overfitting to local changes, resulting in jagged fluctuations in the prediction results.

The GBDT model has significantly improved stability compared to the MLP model. By adopting the ensemble learning strategy and iteratively correcting the errors of the previous decision tree, GBDT effectively reduces the variance of the model. However, as a non-temporal model and lacking feature engineering, it performs worse in terms of prediction accuracy than the Poly-LSTM model.

Poly-LSTM combines the advantages of feature engineering and time series modeling. Polynomial feature extraction helps to linearize the complex relationship between capacitance and liquid level, while the LSTM structure captures the time dependence and inertia of liquid level changes. This combination enables Poly-LSTM to effectively filter out noise and provides the best prediction results among all the compared models.

### 4.3. Error Analysis

**Figure 14:**
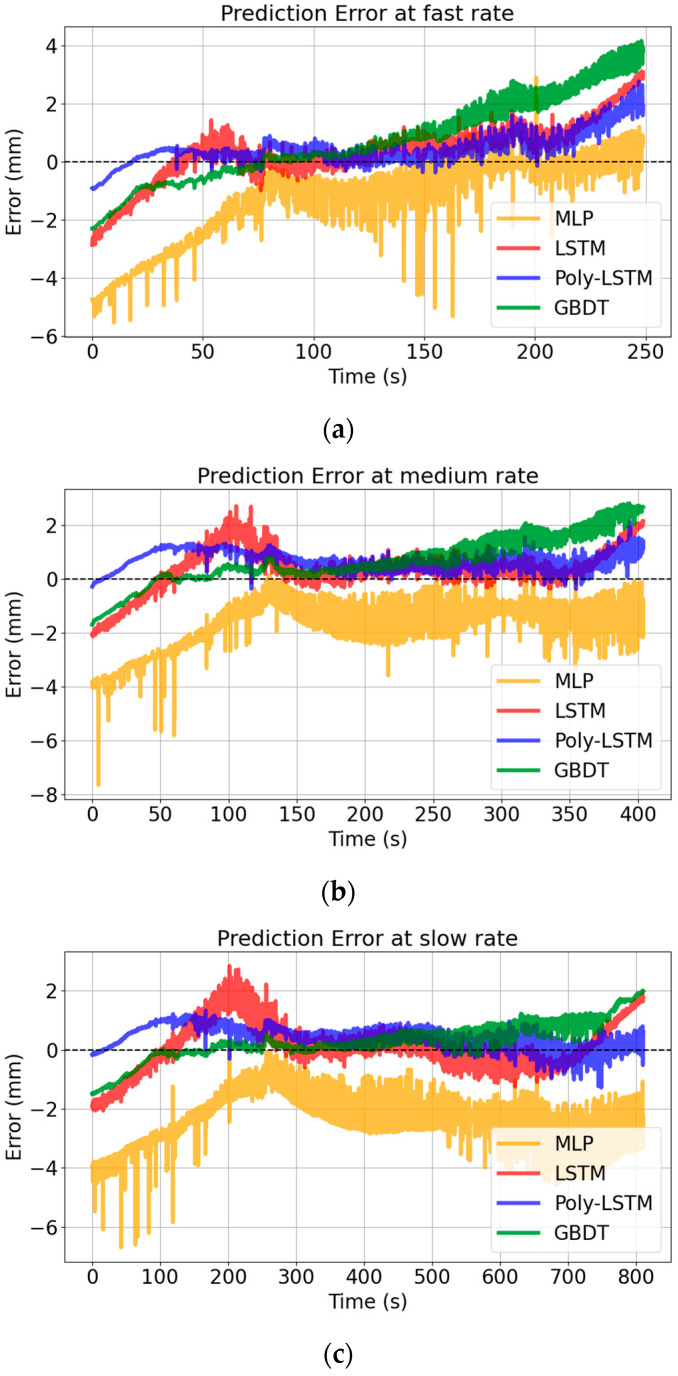

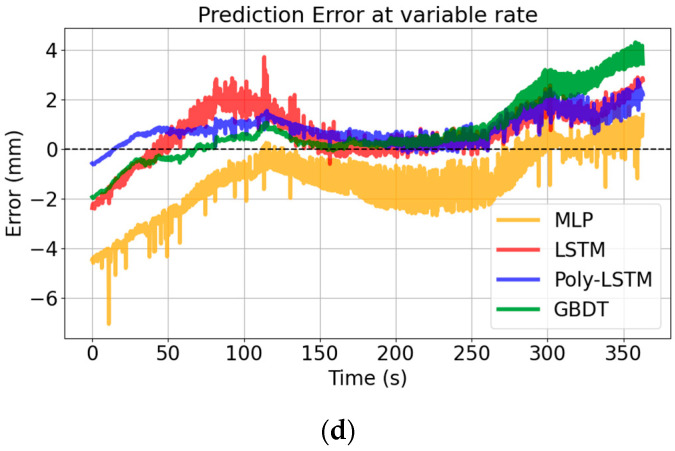
Level estimation errors of different models at different rates. (**a**) Level estimation errors of different models at slow descent rate. (**b**) Level estimation errors of different models at medium descent rate. (**c**) Level estimation errors of different models at fast descent rate. (**d**) Level estimation errors of different models at fast descent rate.

**Figure 15:**
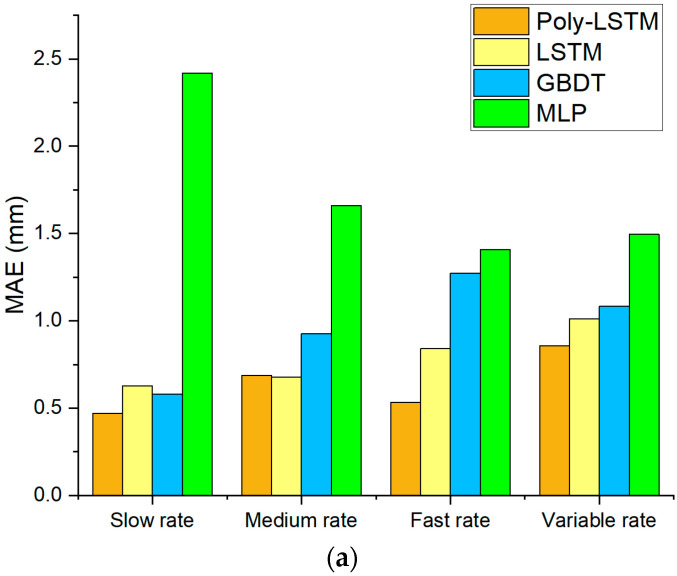

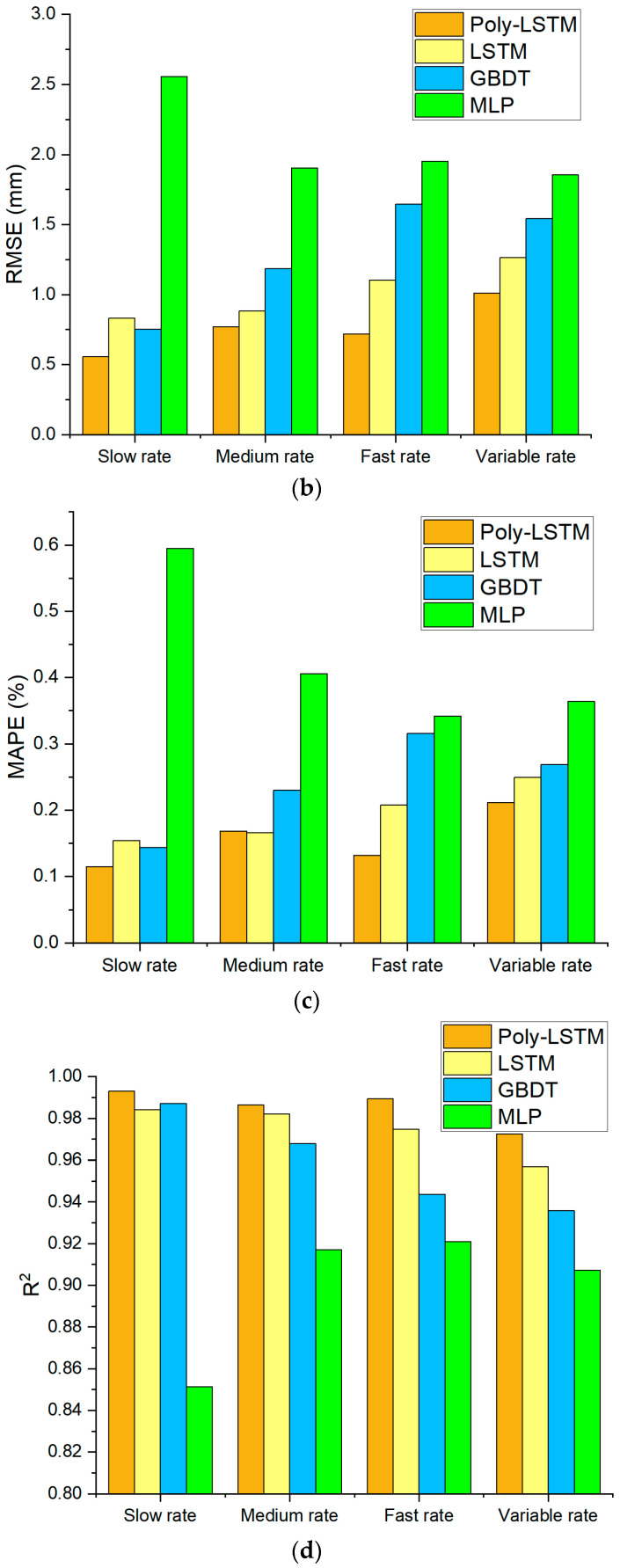
Liquid level estimation results performance index of different models at different descent rates. (**a**) Comparison of MAE for the four models. (**b**) Comparison of RMSE for the four models. (**c**) Comparison of MAPE for the four models. (**d**) Comparison of R^2^ for the four models.

Figure 14 shows that the prediction error distribution of Poly-LSTM is closest to 0, indicating that its predicted values have the smallest deviation from the true values. Different models’ performance indexes of level estimation for the test set are shown in Table 8 using Equations (8)–(11), and the comparison chart of each evaluation index for the four models are shown in Figure 15. It shows that the level estimation indexes of Poly-LSTM are better than other models. The mean absolute error (MAE) is no more than 0.86 mm, the root mean square error (RMSE) is no more than 1.02 mm, and the mean absolute percentage error (MAPE) is no more than 0.22%, respectively. For fast liquid level descent rate measurement, compared with LSTM, the MAE, RMSE, and MAPE decreased by 36.71%, 34.99%, and 36.39% respectively. Therefore, the Poly-LSTM model has strong robustness for liquid level prediction of sulfuric acid with different descent rates.

In addition to prediction accuracy, system latency is another critical performance indicator for dynamic measurement systems. The total latency of the proposed method is determined by the sampling interval of 200 ms. At the fast descent rate of 0.12 mm/s, this latency corresponds to a theoretical level error of approximately 0.024 mm. This value constitutes a small fraction of the overall measured MAE of 0.5319 mm which is 4.5%, indicating that while latency contributes to the dynamic error, the primary improvements of the Poly-LSTM model stem from its ability to accurately compensate for the nonlinear effects of the residual liquid film.

### 4.4. Validation of the Model for Different Environmental Temperatures

**Figure 16:**
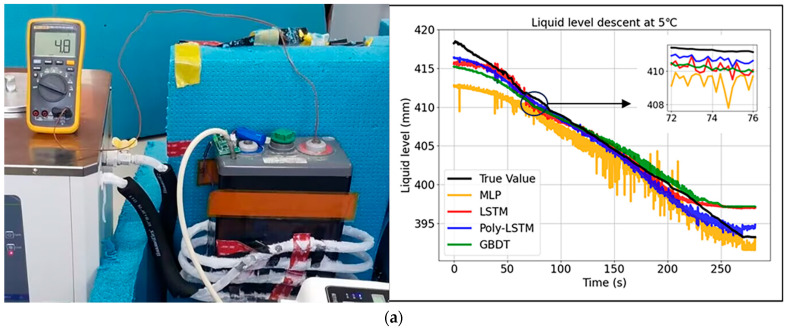

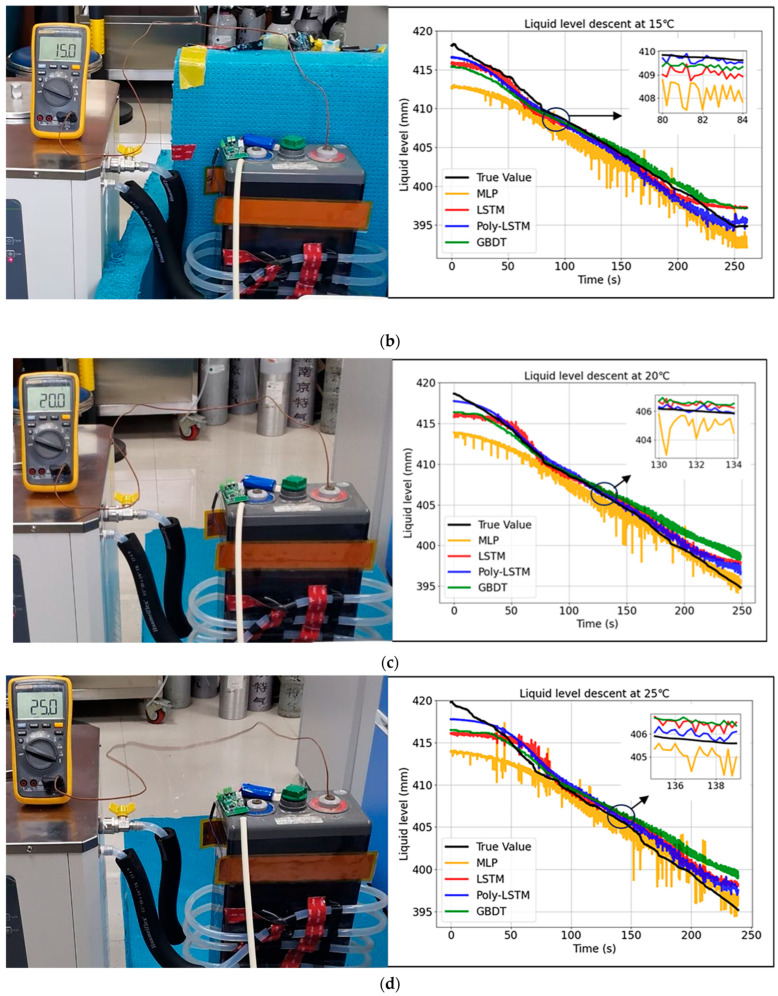
Level estimation results of different models at different environmental temperatures. (**a**) Level estimation results of different models at 5 °C. (**b**) Level estimation results of different models at 15 °C. (**c**) Level estimation results of different models at 20 °C. (**d**) Level estimation results of different models at 25 °C.

All the previous studies were conducted under controlled environmental temperature conditions at 20 °C. However, in actual industrial scenarios, temperature is an important factor that cannot be ignored. To verify the robustness of the liquid level measurement system in this study to different environmental temperatures, the laboratory environment temperature was controlled through an air conditioning system, and experiments were carried out at temperatures of 5 °C, 15 °C, 20 °C, and 25 °C. The rate of liquid level decline remained unchanged, and the liquid level prediction results of the four models under different environmental temperatures are shown in Figure 16. For the non-English terms in the figure, they are just symbol on other bottles which have nothing to do with the study.

**Figure 17:**
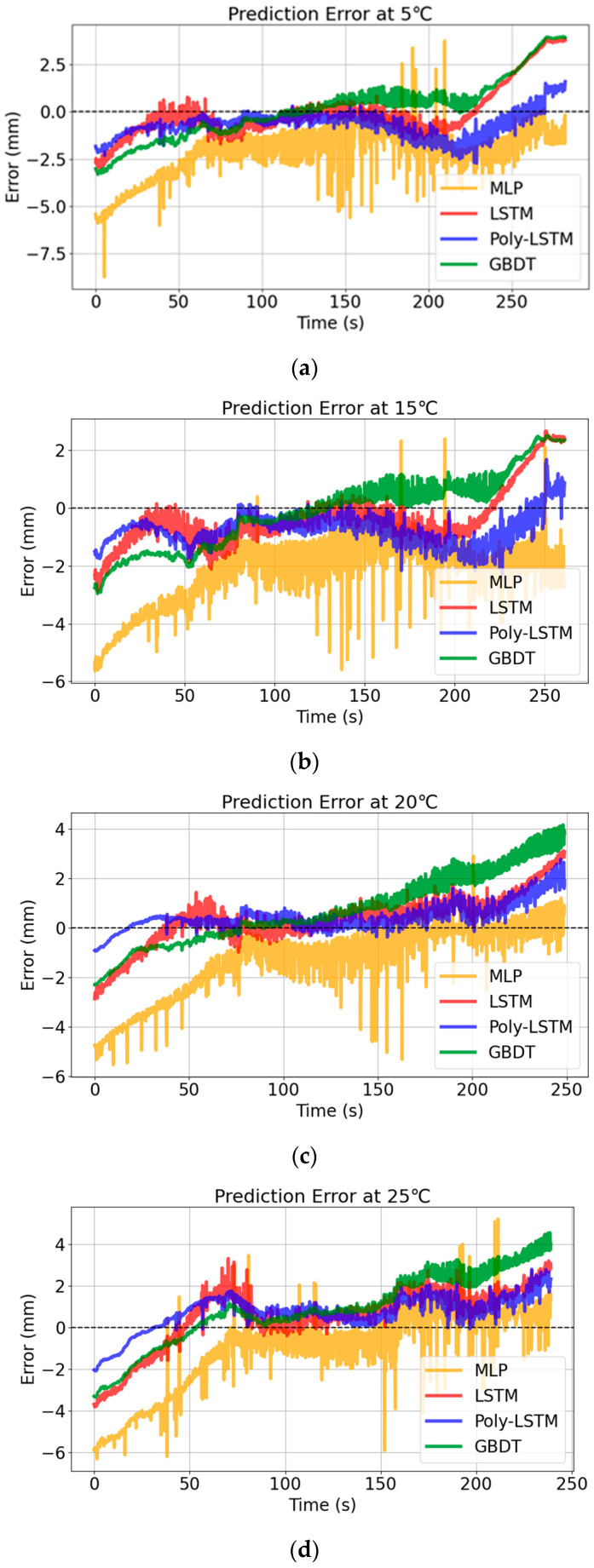
Level estimation errors of different models at different environmental temperatures. (**a**) Level estimation errors of different models at 5 °C. (**b**) Level estimation errors of different models at 15 °C. (**c**) Level estimation errors of different models at 20 °C. (**d**) Level estimation results of different models at 25 °C.

The results show that the prediction curve of Poly-LSTM is the closest to the actual value curve, and the prediction performance under different temperature conditions is relatively good. The prediction errors of the four models are shown in Figure 17.

**Figure 18:**
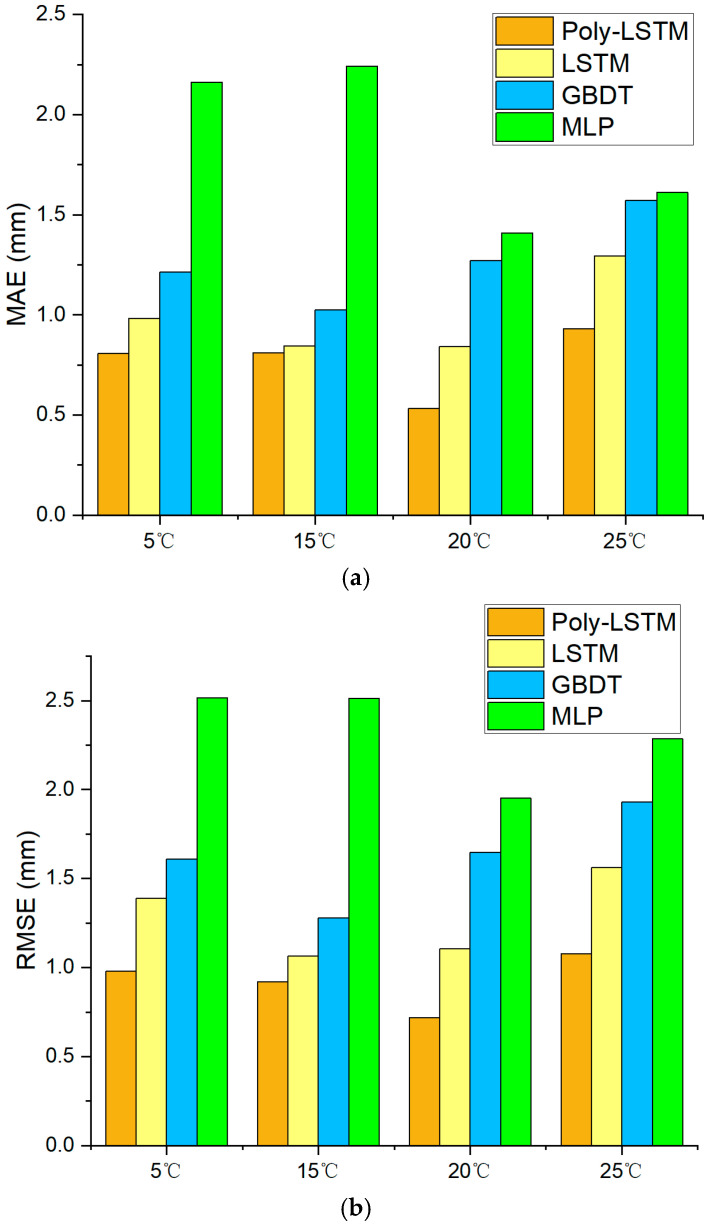

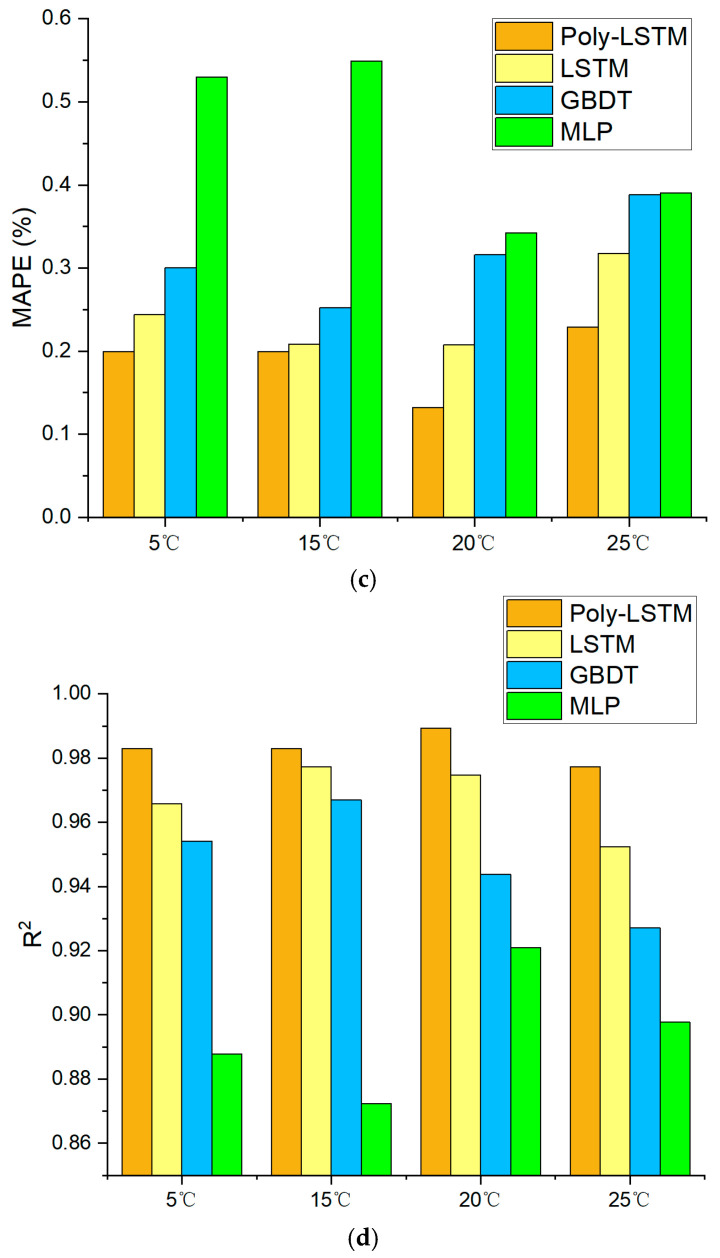
Liquid level estimation results performance index of different models at different environmental temperatures. (**a**) Comparison of MAE for the four models. (**b**) Comparison of RMSE for the four models. (**c**) Comparison of MAPE for the four models. (**d**) Comparison of R^2^ for the four models.

Figure 17 shows that the prediction error distribution of Poly-LSTM under different environmental temperatures is the closest to 0. The deviation between the predicted values of the Poly-LSTM model and the actual liquid level values is very small in the vast majority of cases, indicating that the environmental temperature has a relatively small impact on the capacitance value measurement of the sensor designed in this study. Table 9 and Figure 18 show that the liquid level prediction performance indicators of the four models under different environmental temperatures. The results show that the liquid level prediction indicators of Poly-LSTM are superior to those of the static linear model and MLP. The average absolute error (MAE) is no more than 0.94 mm, the root mean square error (RMSE) is no more than 1.08 mm, and the average absolute percentage error (MAPE) is no more than 0.23%. Taking an environmental temperature of 25 °C as an example, compared with LSTM, MAE, RMSE, and MAPE have decreased by 26.88%, 29.66%, and 26.66%, respectively. Therefore, the Poly-LSTM model has strong robustness in predicting the electrolyte liquid level of lead-acid batteries under different environmental temperatures.

To contextualize the model’s performance, the study first established the system’s practical resolution requirement. The level of electrolyte should be maintained within the upper and low line marks. The operational range between the upper and lower-level marks on the battery was measured to be approximately 35 mm. Applying the common ‘ten-to-one’ engineering principle, this physical tolerance dictates a practical measurement resolution of at least 3.5 mm for effective monitoring. The proposed Poly-LSTM model demonstrated a mean absolute error (MAE) of no more than 0.94 mm across all dynamic test conditions. This achieved precision is more than three times better than the derived practical requirement, confirming its suitability not only for timely alarming but also for providing the high-fidelity data needed for accident analysis in safety-critical applications.

## 5. Conclusions

This paper proposes a hybrid machine learning model (Poly-LSTM) for dynamic liquid level detection of lead-acid battery electrolyte based on a planar capacitor sensor. This model can operate stably under dynamic measurement conditions with different ambient temperatures and different liquid level change rates, and the calculation results are closer to the true liquid level value than linear models, LSTM, GBDT, MLP, and other methods. The input parameters for this model are the capacitance value and the rate of change in capacitance, both of which are easily obtained from capacitance detection of the copper sheets attached to the outer wall of the battery. The model is used as follows. Firstly, the capacitance values at different liquid level descent rates are measured. The capacitance value and capacitance change rate at each sampling interval are taken as base features. Secondly, these features are processed by a Polynomial Feature generator to create high-order and interaction features. These new features explicitly model the complex nonlinear interactions between the inputs and provide a richer, 10-dimensional feature set for the deep learning model. This feature set is then restructured into sequences and fed into an LSTM network to learn the temporal dependencies. Finally, the hidden state of the last time step, which serves as a comprehensive summary of the input sequence, is passed through a fully connected output layer to achieve accurate liquid level prediction. It is found that the MAE of Poly-LSTM algorithm is no more than 0.86 mm, RMSE is no more than 1.02 mm, and MAPE is no more than 0.22%, respectively, which is significantly better than other comparison models. This method fully combines the advantages of polynomial feature generation and LSTM, solves the measurement error caused by the residual liquid film adhesion layer effect, and improves the accuracy of dynamic measurement of lead-acid battery electrolyte level.

Although this study validates the effectiveness of the proposed dynamic measurement method, some challenges remain in applying it to real-world industrial environments such as nuclear power plants. Firstly, electromagnetic interference (EMI) in industrial environments is a problem. Although the FDC2214 chip itself adopts an anti-EMI architecture, future research still needs to include advanced digital filtering algorithms. Secondly, sensor aging due to long-term use may cause measurement drift. This can be solved by developing online self-calibration or drift compensation algorithms, such as using reference electrodes. Finally, when extending this system to multi-cell configurations, relying solely on the Bluetooth module to transmit data to the serial port debugging assistant has significant limitations. Future work will develop a corresponding software control platform to centrally manage a large number of sensors.

## Figures and Tables

**Table 1:** Composition of the experimental data.

Experimental Condition	Descent Rate (mm/s)	Purpose	Number of Runs	Total Sample Points
Quasi-Static Calibration	0.017 (15 rpm)	Linear Model Train	1	7744
Slow Descent	0.035 (30 rpm)	ML Model Train	3	12,361
		ML Model Test	1	4074
Medium Descent	0.07 (60 rpm)	ML Model Train	3	5149
		ML Model Test	1	2041
Fast Descent	0.12 (100 rpm)	ML Model Train	3	3032
		ML Model Test	1	1266
Variable rates	30 rpm–100 rpm	ML Model Test	1	1836
Environmental temperature: 5 °C	100 rpm	ML Model Test	1	1432
Environmental temperature: 15 °C	100 rpm	ML Model Test	1	1327
Environmental temperature: 25 °C	100 rpm	ML Model Test	1	1217
Total			17	41,479

**Table 2:** Liquid level estimation results performance index of static linear model at different descent rates.

Rotate Speed (rpm)	MAE (mm)	RMSE (mm)	MAPE (%)	R^2^
Slow rate	1.4482	1.6298	0.36	0.8989
Medium rate	2.0705	2.1759	0.51	0.8040
Fast rate	3.1439	3.2622	0.78	0.6118

**Table 3:** Hyperparameters of different models.

Model	Hyperparameter	Search Space	Brief Description
MLP	Hidden layer nodes	[(64, 32), (128, 64), (256, 128, 64)]	Structure of the hidden layers (neurons per layer).
	Learning rate	[0.1, 0.01, 0.001]	Initial learning rate for the optimizer.
	Dropout	[0.2, 0.3, 0.5]	Dropout rate for regularization.
	Maximum iteration	[400, 500, 600]	Number of training epochs.
LSTM	Hidden size	[16, 32, 64]	Dimensionality of the LSTM hidden state.
	Number of layers	[1, 2, 3]	Number of stacked LSTM layers.
	Dropout	[0.1, 0.2, 0.3]	Dropout rate applied between layers.
	Learning rate	[0.01, 0.005, 0.001]	Optimizer learning rate.
	Sequence length	[10, 20, 50]	Input window size (time steps) for sequence data.
	Maximum iteration	[100, 150, 200]	Number of training epochs.
GBDT	Number of Estimators	[50, 100, 200]	The number of boosting stages to perform.
	Learning Rate	[0.01, 0.05, 0.1]	Shrinks the contribution of each tree.
	Maximum Depth	[3, 4, 5]	The maximum depth of the individual regression estimators.

**Table 4:** Basic parameter values of the Poly-LSTM.

Parameter	Value
Number of layers	1
Input layer nodes	10
Hidden size	64
Output layer nodes	1
Learning rate	0.01
Maximum iteration	200
Dropout	0.3
Sequence length	20

**Table 5:** Basic parameter values of the MLP.

Parameter	Value
Input layer nodes	2
Hidden layer nodes	Hidden layer 1: 256 Hidden layer 2: 128 Hidden layer 3: 64
Output layer nodes	1
Learning rate	0.1
Maximum iteration	600
Dropout	0.2

**Table 6:** Basic parameter values of the LSTM.

Parameter	Value
Number of layers	1
Input layer nodes	2
Hidden size	64
Output layer nodes	1
Learning rate	0.01
Maximum iteration	200
Dropout	0.3
Sequence length	20

**Table 7:** Basic parameter values of the GBDT.

Parameter	Value
Number of Estimators	200
Learning Rate	0.01
Maximum Depth	5

**Table 8:** Liquid level estimation performance index of different models at different descent rates.

Liquid Level Descent Rate	Algorithm	MAE	RMSE	MAPE (%)	R^2^
Slow rate	Poly-LSTM	0.4702	0.5564	0.1149	0.9930
	LSTM	0.6285	0.8326	0.1539	0.9842
	GBDT	0.5795	0.7536	0.1435	0.9871
	MLP	2.4190	2.5565	0.5947	0.8513
Medium rate	Poly-LSTM	0.6857	0.7688	0.1683	0.9865
	LSTM	0.6785	0.8825	0.1663	0.9822
	GBDT	0.9265	1.1855	0.2300	0.9678
	MLP	1.6593	1.9029	0.4057	0.9170
Fast rate	Poly-LSTM	0.5319	0.7180	0.1320	0.9893
	LSTM	0.8404	1.1045	0.2075	0.9747
	GBDT	1.2719	1.6464	0.3157	0.9437
	MLP	1.4093	1.9514	0.3420	0.9210
Variable rate	Poly-LSTM	0.8572	1.0117	0.2115	0.9724
	LSTM	1.0123	1.2637	0.2490	0.9569
	GBDT	1.0823	1.5430	0.2685	0.9358
	MLP	1.4944	1.8541	0.3639	0.9073

**Table 9:** Liquid level estimation performance index of different models at different environmental temperatures.

Environmental Temperature	Algorithm	MAE	RMSE	MAPE (%)	R^2^
5 °C	Poly-LSTM	0.8076	0.9794	0.1994	0.9830
	LSTM	0.9832	1.3880	0.2440	0.9658
	GBDT	1.2154	1.6092	0.3004	0.9541
	MLP	2.1610	2.5156	0.5296	0.8878
15 °C	Poly-LSTM	0.8098	0.9195	0.1996	0.9829
	LSTM	0.8458	1.0639	0.2085	0.9772
	GBDT	1.0263	1.2781	0.2522	0.9670
	MLP	2.2409	2.5139	0.5491	0.8724
20 °C	Poly-LSTM	0.5319	0.7180	0.1320	0.9893
	LSTM	0.8404	1.1045	0.2075	0.9747
	GBDT	1.2719	1.6464	0.3157	0.9437
	MLP	1.4093	1.9514	0.3420	0.9210
25 °C	Poly-LSTM	0.9305	1.0786	0.2290	0.9772
	LSTM	1.2954	1.5615	0.3178	0.9523
	GBDT	1.5734	1.9299	0.3886	0.9271
	MLP	1.6111	2.2860	0.3904	0.8977

## Data Availability

The code and data are submitted in the GitHub website publicly. Readers can visit them on https://github.com/PengXu-Hub/POLY-LSTM (accessed on 4 January 2026).
